# Molecular mechanisms of embryonic tail development in the self-fertilizing mangrove killifish *Kryptolebias marmoratus*

**DOI:** 10.1242/dev.199675

**Published:** 2021-12-24

**Authors:** Hussein A. Saud, Paul A. O'Neill, Yosuke Ono, Bas Verbruggen, Ronny Van Aerle, Jaebum Kim, Jae-Seong Lee, Brian C. Ring, Tetsuhiro Kudoh

**Affiliations:** 1 Biosciences, College of Life and Environmental Sciences, University of Exeter, Exeter EX4 4QD, UK; 2 Living Systems Institute, University of Exeter, Exeter EX4 4QD, UK; 3 Cefas Weymouth Laboratory, International Centre of Excellence for Aquatic Animal Health, Weymouth DT4 8UB, UK; 4 Department of Biomedical Science and Engineering, Konkuk University, Seoul 05029, South Korea; 5 Department of Biological Sciences, College of Science, Sungkyunkwan University, Suwon 16419, South Korea; 6 Department of Biology, College of Science and Math, Valdosta State University, 1500 N. Patterson St., Valdosta, GA 31698, USA

**Keywords:** Selfing, Isogenic, Forward genetics, Tail bud, Noto, Mesogenin, Mutant, RNA-seq

## Abstract

Using the self-fertilizing mangrove killifish, we characterized two mutants, *shorttail* (*stl*) and *balltail* (*btl*). These mutants showed abnormalities in the posterior notochord and muscle development. Taking advantage of a highly inbred isogenic strain of the species, we rapidly identified the mutated genes, *noto* and *msgn1* in the *stl* and *btl* mutants, respectively, using a single lane of RNA sequencing without the need of a reference genome or genetic mapping techniques. Next, we confirmed a conserved morphant phenotype in medaka and demonstrate a crucial role of *noto* and *msgn1* in cell sorting between the axial and paraxial part of the tail mesoderm. This novel system could substantially accelerate future small-scale forward-genetic screening and identification of mutations. Therefore, the mangrove killifish could be used as a complementary system alongside existing models for future molecular genetic studies.

## Abstract (precis)

**Summary:** The mangrove killifish, a self-fertilizing vertebrate model, allows rapid identification of the mutated genes *noto* and *msgn1* from mutant strains and their genetic roles in tail bud development.

## INTRODUCTION

Within vertebrate species, the embryo is organized as a head, trunk and tail along the anterior to posterior axis. Although the trunk and tail consist of a common set of tissues, including notochord, somites and neural tube (spinal cord), the timing and location of development of the trunk and tail have fundamental differences (Goto et al., 2017; Attardi et al., 2018). For instance, in zebrafish, the trunk cell fates are specified at gastrula stage around the blastoderm margin where the dorsal-most area gives rise to the notochord, and the lateral side to trunk somites and spinal cord (Kimmel et al., 1990; Woo et al., 1995; Kudoh et al., 2004). At this stage, the cells for tail somites and spinal cord are maintained along the ventral side of the embryo (Kudoh et al., 2004). At the end of the gastrula stage, axial mesoderm cells from the dorsal side and the ventro-lateral mesoderm/ectoderm cells merge to each other and form the tail bud (Kanki and Ho, 1997; Kudoh et al., 2004; Row et al., 2011). The tail bud contains an organizing activity that can promote development of tail axial and non-axial mesoderm and neural ectoderm (Goto et al., 2017; Attardi et al., 2018). Although the fundamental role of the tail bud may be conserved in all vertebrate animals, due to their differences in embryonic morphology and size, gene expression patterns and the mechanisms by which the tail bud regulates tail tissue specification and patterning vary depending on the species (Finch et al., 2010; Mourabit et al., 2014).

Here, we introduce a new model species, the self-fertilizing mangrove killifish *Kryptolebias marmoratus*, as a tool for studying gene functions in the tail bud. *K. marmoratus* adult fish are mainly self-fertilizing hermaphrodites with smaller numbers of male fish. As the same mutated DNA sequence (allele) would be inherited by an individual F_1_ fish in the ovotestis (ovary and testis located next to each other) (Camacho Grageda et al., 2004; Sakakura et al., 2006), recessive zygotic mutant phenotypes may be observed in the F_2_ generation derived from a single self-fertilizing F_1_ parent. This makes the process of mutant screening one generation shorter than other dimorphic animal models and omits the process of identifying families carrying a mutant allele, leading to quicker mutant screens using smaller numbers of fish and tanks. Using the mangrove killifish, Ring's group conducted a pilot screen for zygotic lethal mutants by N-ethyl-N-nitrosourea (ENU)-induced mutagenesis (Moore et al., 2012), followed by a continued F_3_ screen to confirm zygotic lethal alleles (old and new) and uncover sterile mutant lines (Sucar et al., 2016). From these lines, we selected two mutants, R109/*shorttail* and R228/*balltail*, characterized by their unique phenotypes during tail development. Taking advantage of the small number of polymorphisms found in these inbred self-fertilizing animals, we needed to sequence only a small number of mutant fish embryos using one lane of RNA sequencing (RNA-seq) to identify the key mutations that cause the *stl* and *btl* phenotypes.

Our results provide insights of evolutionary diversification of gene function, in addition to redundancy and specification that facilitate the establishment of different gene and tissue functions during embryonic development. This work also demonstrates the mangrove killifish as a powerful genetic model that could be used for generating mutant lines quickly, characterizing novel phenotypes and identifying mutated genes, thereby contributing to our understanding of the genetic mechanisms underlying embryo development and many other phenotypes.

## RESULTS

### Phenotypes of mutations

**Fig. 1.**
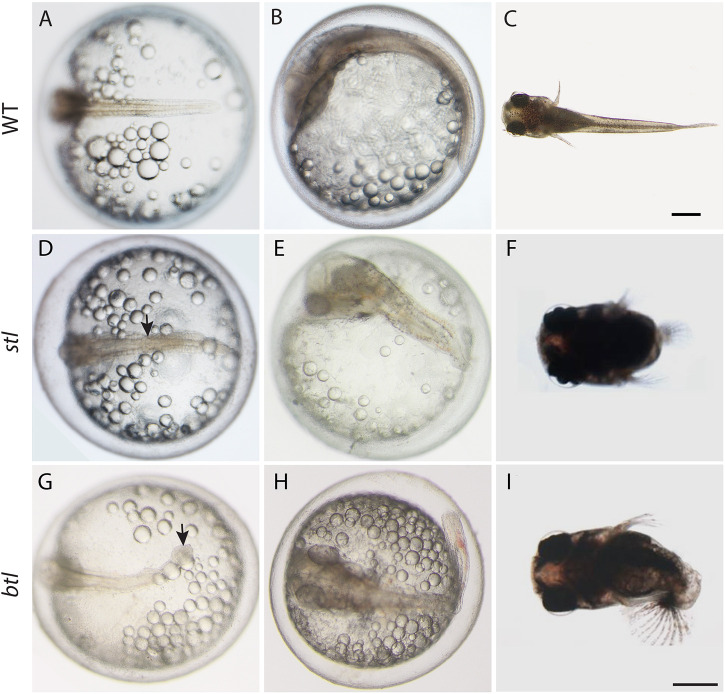
**Morphology *of K. marmoratus stl* and *btl* mutants.** (A-I) WT (A-C), *stl* mutant (D-F) and *btl* mutant (G-I) live embryos at stage 23 (A,D,G), stage 27 (B,E,H) and 1 day post-hatching (C,F,I). Arrow in D indicates the narrow region between the trunk and tail part showing disappearance of notochord along posterior part in the *stl* mutant. Arrow in G indicates the enlarged part of the tail in the *btl* mutant. Scale bars: 200 µm.

To uncover mechanisms of tail development in the self-fertilizing *K. marmoratus*, two ENU-mutated lines, R109 and R228 (Sucar et al., 2016), were analysed. R109/shorttail (*stl*) exhibits reduced tail growth with characteristic narrowing at the trunk-tail junction (Fig. 1D) whereas R228/balltail (*btl*) was characterized by a swollen part at the end of tail resembling a ball shape (Fig. 1G). Both mutations show different phenotypes at late stages of development. In *stl*, the tail becomes shorter during embryonic development and later the posterior part completely disappears (Fig. 1E,F). In contrast, in the *btl* mutants during later embryonic development [stage (St.) 26] the tail forms through a randomly occurring abnormal turn in the anterior and/or posterior tail resulting in irregular tail morphology (Fig. 1H,I). These phenotypes appeared with Mendelian ratios consistent with the recessive nature of a mutation occurring in one gene for each line as they originated from different founding F_0_ mutated fish (Sucar et al., 2016).

### *In situ* hybridization of gene markers in tail tissues at early stages of *K. marmoratus* embryonic development

**Fig. 2.**
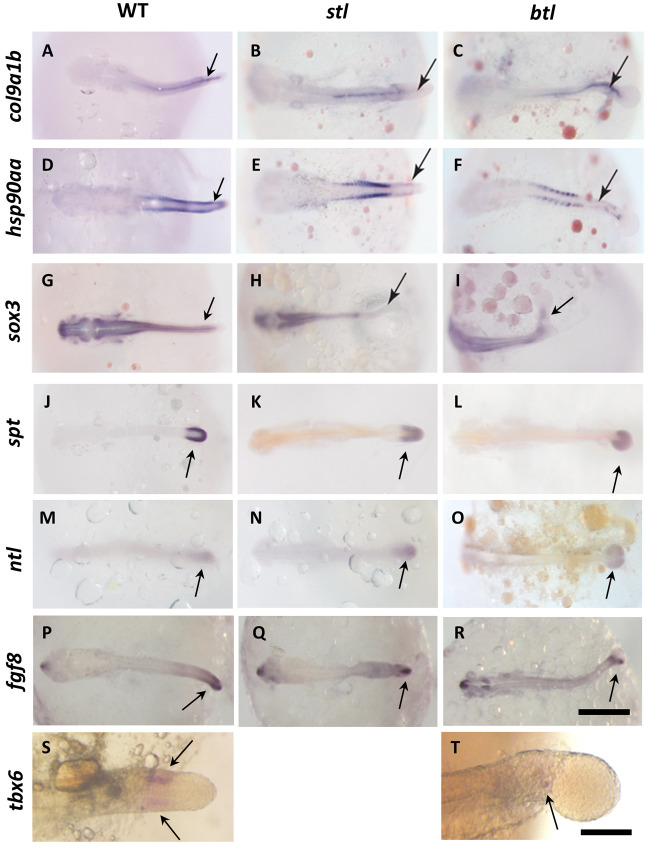
***In situ* hybridization in *K. marmoratus*.** (A-T) Expression of *col9a1b* (A-C), *hsp90aa* (D-F), *sox3* (G-I), *spt* (J-L), *ntl* (M-O), *fgf8* (P-R) and *tbx6* (S,T) in WT, *stl* mutant and *btl* mutant embryos at St. 22/23. Arrows indicate the gene expression domain in the tail. Scale bar: 200 µm (A-R); 50 μm (S,T).

To examine the mechanisms of tail developmental defects in the *stl* and *btl* mutants, seven molecular markers expressed in different domains of the tail tissue were visualized using *in situ* hybridization (Fig. 2). In the *stl* mutant, a notochord marker (*col9a1b*) was not expressed in the tail region (Fig. 2B) suggesting a defect in the tail notochord development in the *stl* mutant. By contrast, *btl* showed slightly expanded expression of *col9a1b* (Fig. 2C). *hsp90aa* was used as a marker for the somite muscle. Our *in situ* staining results revealed loss of expression of *hsp90aa* specifically in the tail part in both *stl* and *btl* mutants (Fig. 2E,F). *sox3* was used as a marker to investigate the effect of the mutations on the neural tube (Mourabit et al., 2014). *stl* mutants showed suppression of *sox3* expression in the tail spinal cord (Fig. 2H). In the *btl* mutant, *sox3* was not clearly affected, although the shape of the expression domain was altered possibly as a result of the bent tail phenotype (Fig. 2I). These data demonstrate that, even though all three marker genes are expressed throughout the trunk and tail, the tail part of the gene expression profiles were primarily suppressed by these mutations, suggesting that the molecular mechanisms of gene regulation in the trunk and tail are different. *spt* is expressed in the tail bud, especially in the paraxial domain and undifferentiated marginal area in the tail bud (Fig. 2J). *spt* expression in the *btl* and *stl* was not significantly altered (Fig. 2K,L). *ntl* is broadly expressed in the wild-type (WT) tail bud (Fig. 2M). *ntl* expression was not significantly altered in the *stl* and *btl* embryos (Fig. 2N,O). Consistent with this, *fgf8* expression in the tail bud (Fig. 2P) was not suppressed in these mutants (Fig. 2Q,R). By contrast, *tbx6* is expressed in the developing WT somite (Fig. 2S) but expression is greatly reduced in the *btl* mutant (Fig. 2T), which is consistent with the reduction of the later somite marker *hsp90aa* (Fig. 2F). All of these *in situ* staining results were consistent in each individual observed (*n*=5).

### Identification of the key mutations of R109/*stl* and R228/*btl* in *noto* and *msgn1*, respectively

**Fig. 3.**
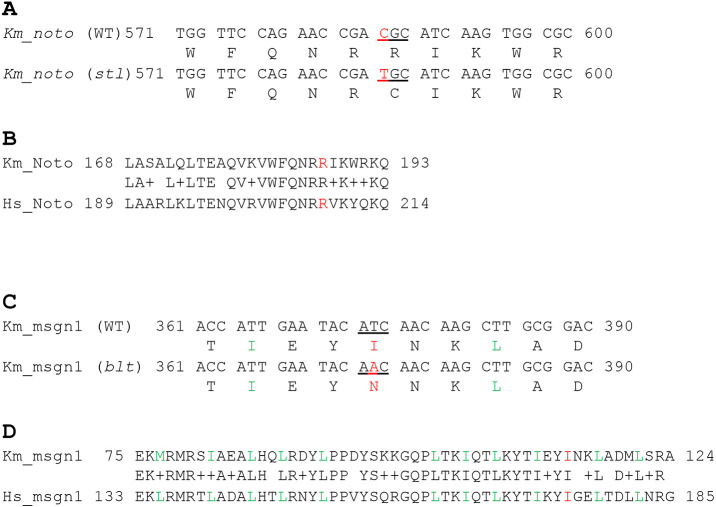
**R109/*stl* and R228/*btl* have a mutation in *noto* and *msgn1*, respectively, in a highly conserved amino acid region*.*** (A) cDNA and protein sequence of *Km_noto* from the Hon9 and *stl* mutant showing amino acid substitution from arginine 187 to cysteine. (B) This arginine is highly conserved in other vertebrate orthologues, including human. (C) cDNA and protein sequence of *Km_msgn1* from the Hon9 and *btl* mutant showing amino acid substitution from isoleucine 117 to asparagine. (D) This isoleucine is highly conserved in other vertebrate orthologues, including human.

To identify the key mutations that caused the *stl* or *btl* phenotype, the protein-coding sequences of the embryonically expressed genes were analysed in mutant and sibling groups using RNA-seq with the screening scheme highlighted in Fig. S1. Naturally spawned eggs were collected from the tank of the WT (*Hon9* strain), R109/*stl* or R228/*btl* mutant strains. The eggs were developed to mid somite stage (St.19), when the *stl* or *btl* phenotype is obvious, and were separated into mutant or non-mutant (sibling) groups. Twenty embryos were pooled from each group (five groups: WT, *stl*, *stl*-sibling, *btl* and *btl*-sibling). Total RNA was prepared from each pool, from which tagged libraries were made and analysed on one lane of RNA-seq using Illumina HiSeq2500 100 bp paired end reading (Fig. S1). The cDNA sequence was *de novo* assembled using Trinity v2.2.0. Using the RNA-seq data, all homozygous non-synonymous polymorphic variations were identified by KisSplice (Lopez-Maestre et al., 2016). According to these data, there were 4544 homozygous variants between WT and the R109/*stl* mutants (Table 1, Screen 1). These variants were narrowed down to 91, representing those showing 100% enrichment in the R109/*stl* mutant and 0% in the WT (Table 1, Screen 2). However, most of these variants showed some unnatural patterns, such as a small number of reads from particular samples (e.g. sibling sample or WT sample) or the number of reads of a WT variant being smaller than that for the mutant variant. Therefore, these variants did not follow a Mendelian ratio, suggesting that these variants are not responsible for the mutant phenotype. To remove these unreliable variants, candidate variants were screened with the following further criteria: WT read is more than ten (Table 1, Screen 3); mutant read is more than five (Table 1, Screen 4); sibling read is heterozygous and more than four from each variant (Table 1, Screen 5); and in the sibling read WT variant is more than mutant variant (Table 1, Screen 6). By eliminating variants that did not match these criteria, the candidate mutations of R109/*stl* were successfully narrowed down to one gene, which turned out to be *noto* (Fig. 3, Tables 1, 2). Table 2 shows the pattern of reads from the WT, R109/*stl* and R109 sibling that demonstrated that the mutation in nucleotide (nt) 586 is 100% enriched in the mutant group, 19% in the sibling and 0% in the WT. The *noto* gene in R109/*stl* showed a point mutation in nt586 that alters a C-terminal region, leading to a missense base pair transition from cytosine to thymine that results in an amino acid change of arginine (R187) to cysteine (C) (Fig. 3A). The arginine in this domain is conserved between fish and humans, suggesting its important role and supporting the idea that the mutation of R187C caused compromised function of the *noto* gene (Fig. 3B).

**Table 1.**
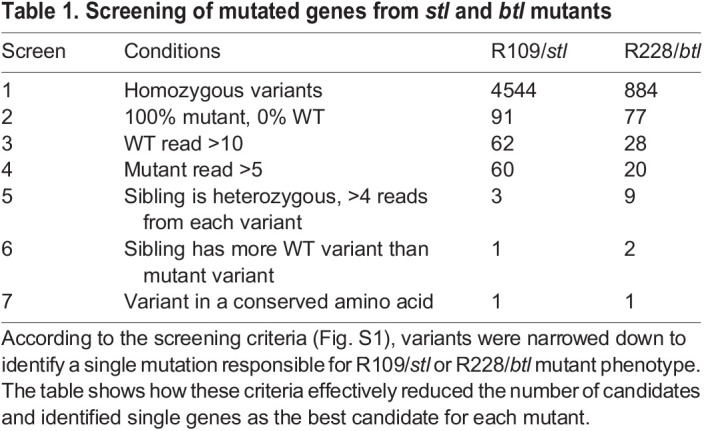
Screening of mutated genes from *stl* and *btl* mutants

**Table 2.**
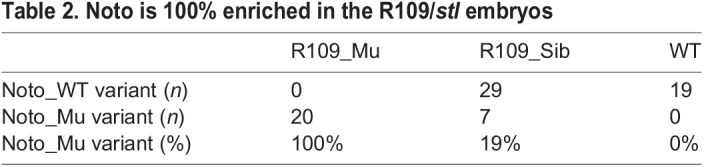
Noto is 100% enriched in the R109/*stl* embryos

Similarly, using the same criteria (Fig. S1) a different mutant allele causing the R228*/btl* phenotype was identified. There were 884 homozygous variants identified between WT and R228/*btl* mutant libraries (Table 1, Screen 1). Among these variants, there were 77 that showed 100% enrichment in the R228/*btl* mutant and 0% in the WT (Table 1, Screen 2). By applying the screening criteria described above (Table 1, Screens 3-6), the candidate variants were successfully narrowed down to two (Table 1). Of these two variants, one is located in the *msgn1* gene at the nt274, resulting in a highly conserved amino acid, isoleucine (I114), being changed to asparagine (N) (Fig. 3C). This isoleucine is a part of the essential structure of the protein, forming a leucine-zipper motif, suggesting that the mutation I114N would cause compromised function of *msgn1* (Fig. 3D). Considering that only one candidate gene fitted our screening conditions 1-7 for *stl* and *btl*, we concluded that *noto* and *msgn1* were very strong candidates for alleles causing the *stl* and *blt* mutant phenotypes, respectively.

In addition to identifying the mutated candidate genes from these mutants, RNA-seq data also provided gene expression profiles in the mutants (Figs S2 and S3). The gene expression level was estimated by coverage and compared between the mutants and siblings. Among the top 30 genes that were downregulated in *stl* and for which expression patterns are known in zebrafish (ZFIN gene database), 15 genes were specific to notochord, four genes to somite muscle, four genes to the CNS and one gene to the heart, and six genes were broadly expressed (Fig. S2). In the case of *btl*, the top 30 downregulated genes were 11 somite muscle-, four notochord-, five CNS- and one epidermis-specific genes and nine broadly expressed genes (Fig. S3). These results further support the suggestion that *stl* and *blt* have primary defects in the notochord and muscle, respectively.

### Blocking *noto* or *msgn1* in medaka phenocopies *stl* or *btl*, respectively

**Fig. 4.**
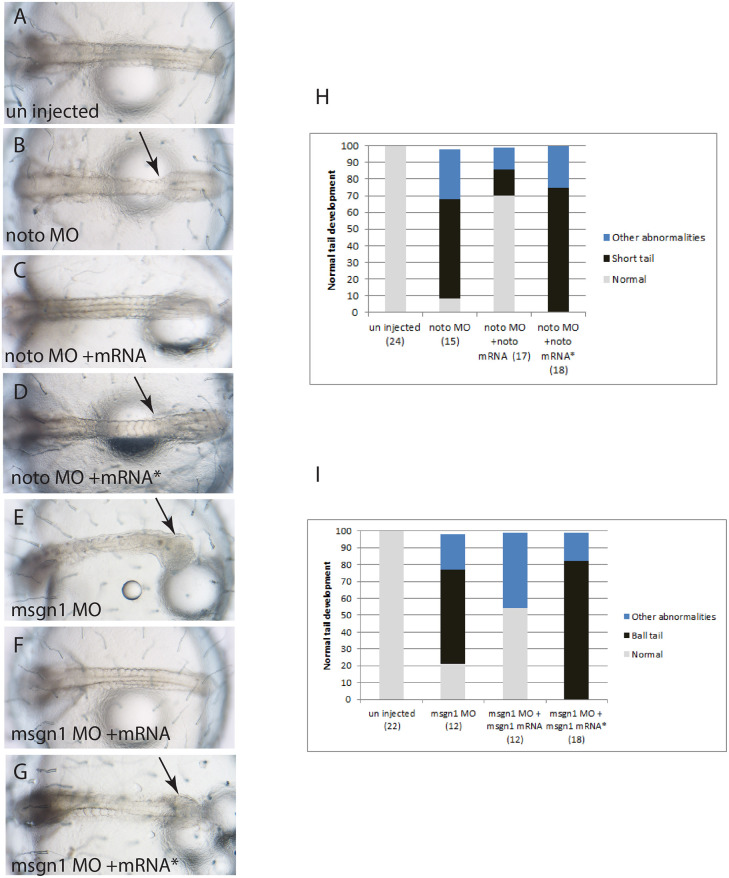
**Medaka *noto* and *msgn1* morphants phenocopy the mangrove killifish *stl* and *btl* mutants, respectively.** (A) WT medaka embryo (St.23). (B) Medaka *noto*MO phenocopies the *stl* mutant (arrow indicates the typical narrowing of the trunk-tail junction). (C,D) The morphant phenotype is rescued by co-injection of the wild-type *Km*_*noto* mRNA (C), but is not rescued by co-injection of mutated form (R187C) of *Km_noto* mRNA (D, arrow). (E-G) Similarly, medaka *msgn1*MO phenocopies the *btl* mutant (E, arrow), and is rescued by wild-type *Km_msgn1* mRNA (F) but not by a mutant (I114N) form of *Km_msgn1* mRNA (G, arrow). (H,I) Histograms showing the proportion of morphant embryos rescued by mRNA injection.

To confirm that the mutation phenotypes of *K. marmoratus* resulted from missense mutant alleles of *noto* and *msgn1* in *stl* and *btl*, respectively, we planned to inject morpholinos (MOs) of these genes to phenocopy the mutant phenotype. However, *K. marmoratus* often hold fertilized eggs within the body and randomly release eggs to the water at varying stages of development. Therefore, it is difficult to obtain many one-cell-stage embryos for MO injections. To overcome this problem, we designed *noto* and *msgn1* MO orthologues to medaka (*Oryzias latipes*) and injected the MO into medaka embryos to phenocopy the *K. marmoratus stl* and *btl* mutant phenotypes. Indeed, these MO injections produced morphants presenting a typical *stl* phenotype (short tail with narrowing of the trunk-tail junction) with *noto*MO (Fig. 4B) and a typical *btl* phenotype (ball-shaped enlarged tip of tail) with *msgn1*MO (Fig. 4E). In addition, co-injection of mRNAs encoding *Km*_*noto* or *Km*_*msgn1* with MOs rescued the phenocopy (Fig. 4C,F,H,I). To test whether the mutant alleles identified in the *noto* and *msgn1* genes in the mutants are non-functional, mRNAs containing the point mutations of *Km_noto* or *Km_msgn1* were synthesized and also co-injected with their corresponding MOs. The mutant phenotype was not rescued in the resulting embryos (Fig. 4D,G-I). These data indicate that mutated forms of *K. marmoratus stl* in *noto* and *btl* in *msgn1* genes are non-functional, indicating that these mutations are responsible for the *K. marmoratus stl* and *btl* mutant phenotypes.

### *Km_Noto* and *Km*_*msgn1* are expressed in the tail bud and interact with each other in a reciprocal manner

**Fig. 5.**
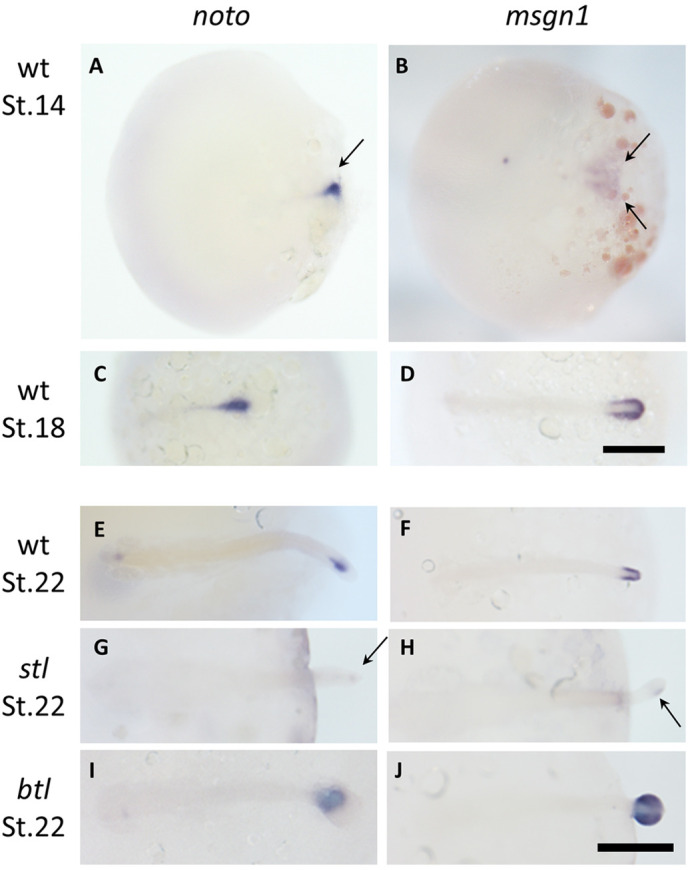
***noto* and *msgn1* expression in WT, *stl* mutant and *btl* mutant embryos.** Dorsal view of WT (A-F), *stl/noto* mutant (G,H) and *btl/msgn1* mutant (I,J) *K. marmoratus* embryos at gastrula or somite stages. Embryos were stained with probes for *noto* (A,C,E,G,I) or *msgn1* (B,D,F,H,J). (A-F) *noto* and *msgn1* are expressed at St.14 (mid gastrula), St. 18 (early somite) and St. 22 (late somite) in the axial (A, arrow) and paraxial (B, arrows) domains of the tail bud, respectively. (G-J) At the late somite stage (St.22), *noto* and *msgn1* expression are both suppressed in the *stl* mutant (G,H) but expanded in the *btl* mutant (I,J). Scale bar: 200 µm.

To examine the expression pattern of *noto* and *msgn1* in *K. marmoratus*, whole-mount *in situ* hybridization was conducted. We found that *K. marmoratus noto* is expressed around the posterior end of the axial mesoderm (Fig. 5A), which gives rise to the axial part of the tail bud and is indeed expressed in the central part of the tail bud, including newly synthesized notochord cells (Fig. 5C,E) whereas *msgn1* is expressed in the posterior paraxial mesoderm from gastrula stage (Fig. 5B) and continues to be expressed in the paraxial part of the tail bud (Fig. 5B,D,F). *In situ* hybridization of *noto* in the *stl/noto(−/−)* mutant exhibited suppression of the gene (Fig. 5G), whereas *noto* expression was enhanced in the *btl/msgn1(−/−)* embryo (Fig. 5I). Similar patterns were observed for *msgn1*; *msgn1* presented reduced expression in the *stl/noto(−/−)* mutant and ectopic expression in *btl/msgn1(−/−)* mutant embryos (Fig. 5H,J).

### Progenitor cells of tail bud behaviour in the medaka *noto* and *msgn1* morphants

**Fig. 6.**
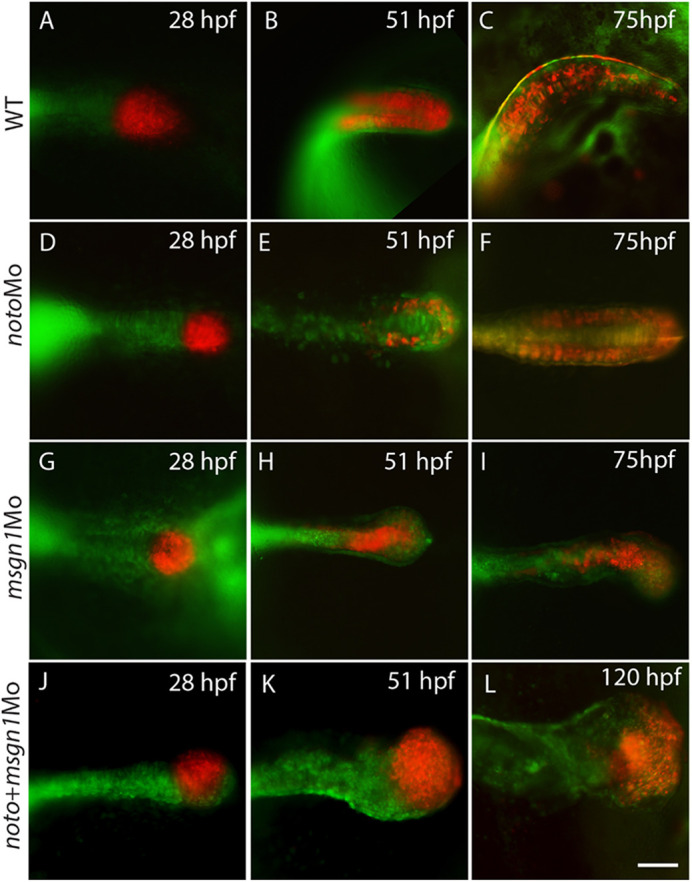
***noto* and *msgn1* morphants show specific migration defects in the tail bud.** Medaka WT (A-C), *noto* morphant (D-F), *msgn1* morphant (G-I) and *noto* and *msgn1* double morphant (J-L) embryos were injected with Kaede mRNA at the one-cell stage. At 28 h post-fertilization (hpf; St.19), tail bud cells were exposed to ultraviolet light to activate the red fluorescence and the cell fate of red-fluorescent cells was examined at subsequent stages. Scale bar: 200 µm.

The mutant phenotype and gene expression data suggest that *noto* and *msgn1* play a crucial role in tail bud development to form axial mesoderm (notochord) and paraxial mesoderm (somite), respectively. To examine cell behaviour of the axial and paraxial part of the tail bud, we used medaka embryos and traced tail bud cell fate in WT and MO-injected embryos. For labelling tail bud cells, Kaede mRNA was injected, which made the embryo fluorescent green. At the tail bud stage, the tail bud was exposed to ultraviolet light, which photoconverted these cells to become fluorescent red (*n*=5 for each morphant). We were thereby able to observe the red cells in the tail bud of WT medaka embryos giving rise to notochord and somite over the next 2 days (Fig. 6A-C). In contrast, in the *noto* morphant medaka embryos, the tail bud cells failed to develop notochord and mainly distributed to the paraxial region (Fig. 6D-F). Conversely, in the *msgn1* morphant, the tail bud cells gathered in the midline and failed to migrate to the paraxial region (Fig. 6G-I). The *noto/msgn1* double MO induced a ball-shaped tail similar to the tail bud as seen in the *msgn1* morphant but with severe failure of the tail bud cell deposition into the axial and paraxial part of the tail (Fig. 6J-L). Though the sample number was small (*n*=5/condition), we observed a consistent pattern in all embryos tested. Collectively, these data indicate that both *noto* and *msgn1* have crucial roles in cell movement and deposition in the tail bud, and therefore reciprocal interaction between these two genes determines a balanced patterning of the tail with respect to axial and paraxial components of the tail tissues.

## DISCUSSION

### Noto maintains the tail organizer activity

We demonstrate here that *stl/noto* mutants exhibit reduced gene expression of tail cell lineage-specific marker genes, including *col9a1b* (notochord), *hsp90aa* (somite) and *sox3* (spinal cord). These data lead to two conclusions. First, although notochord, somite and spinal cord are continuous structures from the trunk to tail, these marker genes expression patterns were primarily suppressed in the tail. This may suggest that molecular and cellular mechanisms of tissue development and associated gene regulation are different in the trunk and tail. Second, these data suggest that Noto is the key regulator for inducing the tail organizer activity that promotes tail notochord development, including cell migration, and may affect other lineages, including somite and spinal cord development. The role of *noto* homologues has been investigated in several model animals, including mice, *Xenopus* and zebrafish, demonstrating that *noto* plays a crucial role in notochord development (Talbot et al., 1995; Halpern et al., 1995; Odenthal et al., 1996; Melby et al., 1996; Abdelkhalek et al., 2004; Yamanaka et al., 2007; Goto et al., 2017). However, from these studies, the role of *noto* in inducing other cell lineages, such as somite, was not clearly determined. Therefore, our *K. marmoratus* mutant data has demonstrated a previously unknown role of *noto* as a key gene regulating other tissues in the tail. In zebrafish, the *noto/floating head* (*flh*) mutant shows notochord defects in both the trunk and tail. However, in the mangrove killifish *noto/stl* mutant, the defect in the notochord was primarily seen in the tail. The differences in loss of function of *noto* phenotypes between *K. marmoratus* and zebrafish model animals may be due to variations of functional redundancies between *noto* and other key regulators for notochord and other tail tissue development, including *foxa2*, *brachyury*, *spt*, *tbx6* and *msgn1* (Amacher and Kimmel, 1998; Yamanaka et al., 2007). Possibly owing to such gene functions, the notochord phenotype in the trunk seems milder than that in the tail. RNA-seq and genome data (http://rotifer.skku.edu:8080/Km) for *K. marmoratus* and *O. latipes* rule out the possibility that *noto* has a paralogue in these animals that could compensate for the phenotype by redundancy. *In situ* hybridization staining of *ntl* and *fgf8* markers showed that these tail bud genes were not suppressed in the *stl/noto* mutation, suggesting that the earliest step of tail bud stem cell formation may not be regulated by *noto* and that a later step involving exit of the tail bud stem cells to the differentiating and migrating state may be regulated by *noto*. Although *msgn1* and *spt* are both expressed in the paraxial part of the *K. marmoratus* tail bud and possibly show some redundant and overlapping functions in paraxial mesoderm development (Yabe and Takada, 2012), gene expression regulatory mechanisms involving *noto*-mediated tail organizing activity are different: in the *stl/noto* mutant, only *msgn1* was suppressed (Fig. 5H), but *spt* was not (Fig. 2K). This indicates that the link between *noto* and *msgn1* is a crucial mechanism in the tail bud for tail paraxial mesoderm development and organization, but neither affects *spt* expression posteriorly.

### *msgn1* primarily regulates tail paraxial mesoderm organization and development

The function of *msgn1* has also been studied in other model animals, including *Xenopus* (Yoon et al., 2000), mice (Yoon and Wold, 2000) and zebrafish (Fior et al., 2012; Yabe and Takada, 2012; Chalamalasetty et al., 2014; Manning and Kimelman, 2015). These data showed its crucial role in somite (muscle) development. However, these data did not show a differential role of *msgn1* between the trunk and tail. Our *in situ* hybridization data from the *btl/msgn1(−/−)* mutant demonstrated a crucial role of *msgn1* in inducing somite gene expression (*hsp90aa*) in the tail but the same gene expression was not clearly suppressed in the trunk. These data suggest that the role of *msgn1* is particularly important in the tail bud region for specifying paraxial mesoderm cell fate and migration of these cells to the paraxial domain but may have a more redundant role in the trunk paraxial mesoderm. It is also worth noting that *msgn1* gene knockdown does not show a clear ball tail phenotype in the zebrafish (Fior et al., 2012; Yabe and Takada, 2012). The phenotype of *msgn1* loss of function is very mild in zebrafish compared with that in the mangrove killifish *stl/noto(−/−)* and medaka *noto*MO. As discussed in the *noto* section above, the differential phenotypes observed in tail regulatory (loss-of-function) genes may be due to variations of redundancies with other regulatory genes. For example, functional synergism and redundancy between *msgn1*, *txb16* and *spt* are crucial for paraxial mesoderm development (Yoon and Wold, 2000; Fior et al., 2012; Yabe and Takada, 2012; Chalamalasetty et al., 2014). Our RNA-seq and genome data for the mangrove killifish also confirmed that there is no paralogue of *msgn1* in the species. Therefore, the differential balance and level of redundancies between these factors may change the relative contribution of each factor during tail paraxial mesoderm development.

### *noto* and *msgn1* are crucial for the migration and deposition of tail bud cells to form notochord and somite, respectively

Although there is an apparent epistatic relationship between the *noto* and *msgn1* genes, they may have independent and primary roles in the regulation of cell migration and localization of the notochord and somite cells, respectively (Yamanaka et al., 2007; Yabe and Takada, 2012). We labelled the tail bud cells at the tail bud stage using Kaede fluorescent protein and traced the tail bud cell fates in control, *noto*MO, *msgn*MO and double MO morphants in medaka embryos. These data reveal specific loss of cells migrating towards notochord or muscle in the *noto*MO and *msgn1*MO, respectively, indicating a crucial role of *noto* and *msgn1* in cell movement and localization. Although *notoMO* cells can still migrate to the somite position, gene expression for the tail somite was suppressed (Fig. 2E), suggesting that *noto* has dual roles in the tail bud, maintaining the tail bud organizer activity to induce key tissues in the tail and, at the same time, promoting the tail bud cells to migrate towards and/or along the midline of the tail. The exclusion of the tail bud cells from the notochord in the Noto knockdown embryo in medaka is consistent with previous results in the *flh* zebrafish mutant and *Noto* knockout mice (Halpern et al., 1995; Melby et al., 1996; Yamanaka et al., 2007). Equally, exclusion of labelled tail bud cells from the differentiated somite in the *msgn1* knockdown in medaka is also consistent with previous reports in mice (Yamanaka et al., 2007); however, our time-course live imaging directly showed a differential and exclusive cell-sorting mechanism by the presence/absence of the *msgn1* gene (Fig. 6). In zebrafish, the *msgn1* knockdown phenotype is much more subtle, possibly owing to higher redundancy with *spt*, therefore such a clear role of *msgn1* in the paraxial mesoderm development on its own was not investigated (Yabe and Takada, 2012). Therefore, our data clarified differential and interactive roles of *noto* and *msgn*1 in cell sorting between axial and paraxial mesoderm in the tail bud with a clear phenotype and effective live-imaging analysis.

Although the severity of abnormalities occurring in the trunk and tail are different in killifish, zebrafish and mice, overall function of *noto* and *msgn1* as an organizer for the axial and paraxial mesoderm would also be conserved. However, possibly owing to some differential genetic redundancies and morphological differences, our data in killifish and medaka species showed an enhanced phenotype in the tail compared with that in zebrafish. This might be partly due to the large yolk in these fish species as epiboly has to migrate a greater distance and therefore formation of the tail bud occurs before the completion of epiboly (Mourabit et al., 2011). If the defect caused by the *noto* mutation leads to failure of a fully functional tail bud formation and if that occurs before the end of epiboly, subsequent tail development may be severely affected. We have previously reported that a more severe phenotype in mangrove killifish than in zebrafish was observed in embryos treated with the Bmp inhibitor dorsomorphin (Mourabit et al., 2014). Dorsomorphin-treated zebrafish embryos can complete epiboly and form a tail bud (Yu et al., 2008). However, in dorsomorphin-treated mangrove killifish embryos, epiboly is delayed, causing premature tail bud formation in multiple locations and the ‘tail islands’ phenotype (Mourabit et al., 2014). This provides supportive evidence that the tail phenotype may be more severe in *K. marmoratus* and medaka compared with zebrafish.

### *K. marmoratus* as a model for mutants and associated gene analyses

This is the first report of the use of *K. marmoratus* as a model for mutant and associated gene analyses. *K. marmoratus* is a very unique self-fertilizing fish. Because mutagenized hermaphrodites give rise to both oocyte and sperm within the same body, the generation of homozygous mutants from single hermaphroditic lineages does not require large amounts of labour, facilitating quick generational screening and simple maintenance of the mutant fish lines in a highly reduced space compared with zebrafish and medaka. Here, we applied a single lane of RNA-seq, including WT, *stl*, *btl* mutant and sibling pools all together, using a simple bioinformatics pipeline and screening criteria for narrowing down the mutations to identify the mutated gene for these two mutants (Fig. S1, Tables 1-3). For this, we did not need to use outcrossing or mapping of genetic loci but only needed to sequence a small number of embryos (e.g. 20 mutant embryos as a pool) without the need for replicates. The success of such a simple sequencing strategy to identify the key mutation(s) is largely due to the character of the inbred mangrove killifish genome (isogeny). Initially, Tatarenkov et al. (2010) identified a series of commonly utilized laboratory strains of the mangrove killifish by microsatellite analysis, including the *Hon9* strain used here for mutagenesis. More recently, many more strains have been identified by whole-genome sequencing and comparison of heterozygosity levels (Lins et al., 2018). In this study, for example, the highly isogenic *Hon9* strain used for mutagenesis and maintained for over 30 years of inbreeding in the laboratory, exhibits 99.97% homozygosity of single nucleotide polymorphisms by next generation rad-tag sequencing (B.C.R., F. Agyabeng-Dadzie and J. F. Elder, unpublished). Consequently, we identified single variant genotypes as the candidate cause of the observed phenotypes in the *noto* and *msgn1* genes, respectively. Therefore, the method that we applied here for identifying mutations would be highly applicable for future research into mutants generated in this vertebrate model. In particular, forward genetics in this species would become powerful when it is used for identifying parental-effect genes. Parental-effect mutant screens require four generations of screening, and maintenance of large numbers of fish until completion of the screening process, making it difficult to screen for mutants at saturation levels. However, by using this self-fertilizing fish, the screening process could be reduced by one generation and therefore large-scale screening would be possible (Sucar et al., 2016). It would also be interesting to examine mutation profiles of subtle, non-lethal phenotypes, as such phenotypes are still relatively under-explored in other model organisms. In the self-fertilizing animal, once such phenotypes are found, the homozygous ‘line’ can be directly obtained from the offspring without having to identify carriers of the same mutation from two sexes. Therefore, the process of breeding and analyses would become highly simplified.

**Table 3.**
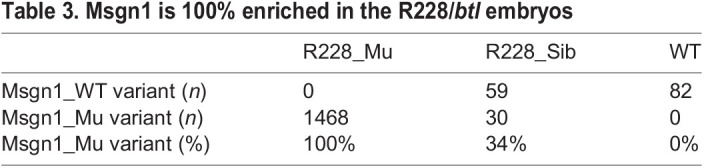
Msgn1 is 100% enriched in the R228/*btl* embryos

Although it is difficult to obtain many one-cell-stage *K. marmoratus* embryos owing to internal self-fertilization, we show here that it is possible to use medaka to confirm mutants by MO and mRNA injection analyses. The medaka rice fish was previously categorized as killifish and is indeed genetically close to the mangrove killifish. The morphology, size and developmental pattern of the embryos are similar (Mourabit et al., 2011; Iwamatsu, 2004). The genome size is similar (700 Mb for the mangrove killifish and 800 Mb for medaka). Cross-species *in situ* hybridization is possible between these two species but not with zebrafish (e.g. *ntl* probe in this study). Thus, the novel approach demonstrated here for identifying and analysing mutants and mutated genes offers an interesting possibility of further gene discovery and analyses in a variety of areas in genetics, such as developmental biology, epigenetics and behaviour.

## MATERIALS AND METHODS

### Fish husbandry

ENU-induced *K. marmoratus* mutant strains (Moore et al., 2012) and the parental WT strain, *Hon9*, were maintained at constant laboratory conditions, 26°C±1°C, 14-15 ppt salinity, 12 h light:12 h dark photoperiod. Individuals were reared in 1500 cm^3^ plastic containers; live *Artemia* were provided once a day as food for the fish, along with weekly water changes. Eggs of each strain were kept in Petri dishes at 26°C until hatching (12-21 days) and were used to maintain stocks or selected for use as mutants for further experiments.

### *In situ* hybridization

*In situ* hybridization as described by Mourabit et al. (2014) was applied to different stages of embryos depending on the type of gene markers observed. The mangrove killifish gene probes for *in situ* hybridization were designed using the cDNA sequence obtained from the *de novo* assembly of RNA-seq. The *Km_sox3* probe was previously reported (Mourabit et al., 2014). cDNAs for *Km_col9a1b*, *Km_hsp90aa* and *Km_fgf8* were amplified with nested PCR and subcloned into pGMT Easy. *Km_spt*, *Km_noto*, *Km_msgn1*, *Km_ntl* and *Km_tbx6* cDNAs were *in vitro* synthesized by GeneArt (Thermo Fisher).

### RNA-seq transcriptome analysis

RNeasy Mini Kits (Qiagen) were used to extract total RNA from 20 embryos (St.16-18) for each strain: WT progenitor (*Hon9*), *btl*, *stl* and their non-mutant siblings. RNA quality of samples was confirmed using an Agilent RNA 6000 Nano Kit before moving forward with the sequencing process. Sequencing libraries were prepared using the RNA-seq directional protocol (Illumina) and sequenced in one lane of an Illumina HiSeq 2500 v3 next generation sequencer with 100 bp paired end reads.

The sequencing data was first trimmed to remove sequencing adaptors and low-quality terminal ends (<Q20) and then short sequences were removed using fastq-mcf v1.1.2-537 (https://github.com/ExpressionAnalysis/ea-utils). *De novo* transcriptome assembly was performed for each of the groups using Trinity v2.2.0 (Haas et al., 2013). Variants between the groups were identified and quantified using KisSplice v2.4.0-p1 (Lopez-Maestre et al., 2016) with a k-mer size of 53. The variants identified by KisSplice were mapped to the *de novo* transcriptomes with BLASTn v2.5.0 (Altschul et al., 1990) to obtain the associated transcript. The transcripts containing variants were then annotated with BLASTn to NCBI-nr (downloaded 11 November, 2016) with an e-value threshold of 1e^−4^, keeping only the best hit. To identify candidate mutation-related variations, we filtered the list of variations produced by KisSplice with custom scripts, applying the following criteria: 0% of reads in the WT group compared with 100% of reads in the mutant group, with the sibling group being intermediate.

Quantification of gene expression was performed using Salmon (1.5.0) against the coding sequences in the reference assembly ASM164957v2 (Patro et al., 2017). Differential expression was performed using NOISeq (2.28.0) (Tarazona et al., 2012, 2015) comparing mutants with WT and their phenotypically normal siblings.

### MOs and mRNAs

MOs of medaka *msgn1* (5′-ACAGGATTTCAGCTTCCACGTCCAT-3′) and *noto* (5′-CCTGCCTTTGCTGTCCTGTGGATC-3′) were generated by Gene Tools LLC. Capped mRNAs for *Km_noto* (WT), *Km_noto* (*stl* mutant form), *Km_msgn1* (WT), *Km_msgn1* (*btl* mutant form) and Kaede were synthesized using mMessage mMachine SP6 kit (Thermo Fisher Scientific) according to the manufacturer's instructions.

*noto* or *msgn1* MOs (2 µg/µl, 1 nl) were injected into one-cell-stage medaka embryos. For the phenotypic rescue experiment, 1 nl of 25 ng/µl mRNAs were co-injected with *noto* or *msgn1* MOs. For cell lineage analysis, Kaede mRNA (100 ng/µl) was also co-injected with a MO.

### Ethics statement

All experiments were approved and performed in compliance with the regulations of the University of Exeter, Animal Welfare Ethical Review Board.

## Supplementary Material

Supplementary information

Reviewer comments
