# Supplementary material for: Molecular mechanisms of embryonic tail development in the self-fertilizing mangrove killifish Kryptolebias marmoratus
Source: Development. 2021 Dec 24;148(24):dev199675. doi: 10.1242/dev.199675 (PMC8722387; doi:10.1242/dev.199675)
Supplement: Supplementary information [file develop-148-199675-s1.pdf]

Supplementary Materials

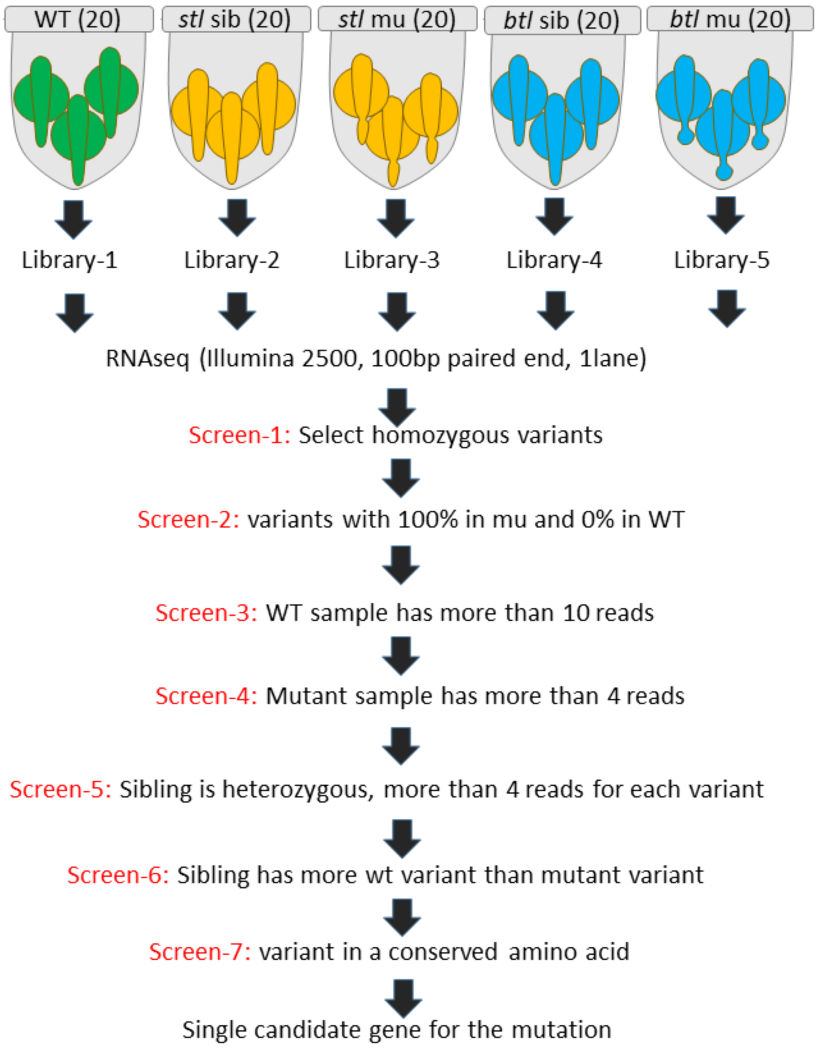

Fig. S1. Identification of mutations from the *stl* and *btl* mutants

Twenty embryos were pooled from WT, R109/*stl* mutants, R109/*stl* siblings, R228/*btl* mutants and R228/*btl* siblings respectively, prepared for generating differently tagged libraries and analysed by single lane of RNAseq. Identified variants were screened by different conditions (screen-1 to 7) to narrow down a single candidate mutation which is responsible for the *stl* or *btl* phenotype.

Development • Supplementary information

	R109sib mean	R109 mean	M	gene	description	expression
1	351.196	2	7.456133	emilin3a	EMILIN-3	notochord
2	67.693	1	6.080935	si:dkeyp-118a3.2	uncharacterized protein si:dkeyp-118a3.2 isoform X1	notochord
3	2890.704	49	5.882495	cmn	calymmin isoform X2	notochord
4	3957.87	81.228	5.606603	col2a1a	collagen	notochord
5	165.741	7	4.565432	loxl5b	lysyl oxidase-like 5b isoform X1	notochord
6	140.299	6.147	4.512478	epha2b	ephrin type-A receptor 2 isoform X1	somite
7	53.764	3.006	4.160724	tcea3	transcription elongation factor A protein 3 isoform X3	somite
8	65.891	4.232	3.96067	ncln	nicalin-1 isoform X4	cns
9	132.243	9.272	3.834167	mybpc1	myosin-binding protein C	notochord
10	472	34	3.79518	epyc	epiphycan	notochord
11	115.077	8.38	3.779505	calua	calumenin-A isoform X1	notochord
12	60.264	4.423	3.768199	scrib	protein scribble homolog isoform X3	broad
13	2296.985	172.349	3.736337	col9a1b	collagen alpha-1(IX) chain isoform X1	notochord
14	347.11	26.186	3.728525	agrn	agrin isoform X4	broad
15	48.169	3.658	3.718978	myo18ab	unconventional myosin-XVIIIa isoform X13	somite
16	1392.613	106.245	3.712328	col2a1b	collagen	notochord
17	133.024	10.515	3.661166	gemin5	gem-associated protein 5 isoform X2	somite
18	104	8.249	3.65622	scn8aa	sodium channel	cns
19	213.152	17	3.648276	loxl1	lysyl oxidase homolog 1	notochord
20	82.945	7.015	3.56364	matn3a	matrilin-3a isoform X3	notochord
21	82.73	7.104	3.541707	tjp1a	tight junction protein ZO-1 isoform X6	notochord
22	213.575	20.3	3.395191	col4a2	collagen alpha-2(IV) chain isoform X2	cns
23	100	10	3.321928	egf	pro-epidermal growth factor	notochord
24	197.033	21.649	3.186065	greb1	protein GREB1 isoform X1	cns
25	858	98.109	3.12852	tfip11	tuftelin-interacting protein 11 isoform X1	broad
26	61.08	7	3.125273	angptl3	angiopoietin-related protein 3 isoform X3	heart
27	105.886	12.217	3.11555	sde2	replication stress response regulator SDE2 isoform X4	broad
28	127.373	15.247	3.062462	stau1	double-stranded RNA-binding protein Staufen homolog 1 isoform X3	broad
29	123.912	14.983	3.047917	pcdh19	protocadherin-19 isoform X5	notochord
30	133.037	16.89	2.977586	otud7b	OTU domain-containing protein 7B isoform X4	broad

Fig. S2. Top 30 genes down-regulated in the R109/*shorttail*

From the RNAseq data, expression was estimated by mean coverage. Expression level is compared between the mutant and sibling samples. The top 30 most highly down-regulated genes which has gene expression reported in zebrafish in zfin database were selected. It is noted that 15 genes among 30 are specifically expressed in the notochord.

Development • Supplementary information

	R228asib mean	R228a mean	M	gene	description	expression
1	780.44	21.235	5.199772	col2a1b	collagen	notochord
2	153.362	4.761	5.009533	xirp1	xin actin-binding repeat-containing protein 1 isoform X1	somite
3	1371.424	47	4.86687	thbs4b	thrombospondin-4-B	somite
4	50	2	4.643856	LOC108243329	mesoderm posterior protein 2-like	somite
5	349.154	18.243	4.258449	igsf9b	protein turtle homolog A isoform X2	somite
6	156.989	8.391	4.225677	gata3	transcription factor GATA-3 isoform X2	epidermis
7	310.484	17.482	4.150577	pum2	pumilio homolog 2 isoform X5	broad
8	201.834	11.765	4.100596	nsd3	histone-lysine N-methyltransferase NSD3 isoform X3	broad
9	129.536	7.896	4.036087	l1cama	neural cell adhesion molecule L1.2 isoform X2	CNS
10	90.233	5.924	3.929012	nob1	RNA-binding protein NOB1 isoform X3	broad
11	205.425	13.582	3.918844	fgfr2	fibroblast growth factor receptor 2 isoform X5	broad
12	275.34	18.34	3.908149	plxnb2b	plexin-B2b isoform X1	CNS
13	208.133	14	3.894007	synpo2la	synaptopodin 2-like protein	somite
14	1754.864	118.555	3.887731	LOC108241321	myosin heavy chain	somite
15	1398.197	96.348	3.859169	col2a1a	collagen	notochord
16	76.147	5.6	3.765288	auts2a	pax	broad
17	160.379	11.949	3.746523	boc	brother of CDO isoform X4	CNS
18	89.803	6.767	3.730175	mybpc1	myosin-binding protein C	somite
19	104.817	8.207	3.674874	myom1b	M-protein	cns
20	95.055	7.587	3.647161	matn4	matrilin-4 isoform X2	notochord
21	350.971	28.389	3.627948	map3k4	mitogen-activated protein kinase kinase kinase 4 isoform X6	Broad
22	195	16	3.60733	myog	myogenin	Somite
23	60.256	4.963	3.601821	qki2	protein quaking-B isoform X1	Broad
24	57.411	4.737	3.599282	myo18ab	unconventional myosin-XVIIIa isoform X13	somite
25	330	27.364	3.592115	tsc1a	TSC complex subunit 1a isoform X1	somite
26	72.08	6.005	3.585363	foxp2	forkhead box protein P2 isoform X2	cns
27	104.596	9.033	3.533479	bcam	basal cell adhesion molecule isoform X4	notochord
28	138.465	11.963	3.53287	pax7a	paired box protein Pax-7a isoform X1	somite
29	87	8	3.442943	LOC108242670	solute carrier family 2	broad
30	143.649	13.328	3.430016	scrib	protein scribble homolog isoform X16	broad

Fig. S3. Top 30 genes down-regulated in the R228/*balltail*

From the RNAseq data, expression was estimated by mean coverage. Expression level is compared between the mutant and sibling samples. The top 30 most highly down-regulated genes which has gene expression reported in zebrafish in zfin database were selected. It is noted that 11 genes among 30 are specifically expressed in the somite muscle.

Development • Supplementary information
