# Supplementary material for: Molecular mechanisms of embryonic tail development in the self-fertilizing mangrove killifish Kryptolebias marmoratus
Source: Development. 2021 Dec 24;148(24):dev199675. doi: 10.1242/dev.199675 (PMC8722387; doi:10.1242/dev.199675)
Supplement: Reviewer comments [file develop-148-199675_review_history.pdf]

Molecular mechanisms of embryonic tail development in the self-fertilizing mangrove killifish *Kryptolebias marmoratus*

Hussein A. Saud, Paul A. O'Neil, Yosuke Ono, Bas Verbruggen, Ronny van Aerle, Jaebum Kim, Jae-Seong Lee, Brian C. Ring and Tetsuhiro Kudoh

DOI: 10.1242/dev.199675

Editor: James Briscoe

Review timeline

Original submission:	31 March 2021
Editorial decision:	5 May 2021
First revision received:	12 October 2021
Accepted:	26 October 2021

Original submission

First decision letter

MS ID#: DEVELOP/2021/199675

MS TITLE: Molecular mechanisms of embryonic tail development in the self-fertilizing mangrove killifish, Kryptolebias marmoratus

AUTHORS: Hussein A Saud, Paul A O'Neil, Yosuke Ono, Bas Verbruggen, Jaebum Kim, Jae-Seong Lee, Brian C Ring, and Tetsuhiro Kudoh

I have now received all the referees' reports on the above manuscript, and have reached a decision. The referees' comments are appended below, or you can access them online: please go to BenchPress and click on the 'Manuscripts with Decisions' queue in the Author Area.

As you will see, the referees express considerable interest in your work, but have some significant criticisms and recommend a substantial revision of your manuscript before we can consider publication. The referees identify several areas where additional clarification is needed or alternative interpretations considered, I would encourage you to address these points. I'd also like to highlight the suggestion of using the RNAseq data to assess whether Kmar has additional Noto or Msgn1 paralogues and to investigate regulatory changes in the mutants. These seem to be constructive proposals. In addition, I agree that the description of the identification of the candidate genes could be condensed and the overall length of the paper reduced.

If you are able to revise the manuscript along the lines suggested, I will be happy receive a revised version of the manuscript. Your revised paper will be re-reviewed by one or more of the original referees, and acceptance of your manuscript will depend on your addressing satisfactorily the reviewers' major concerns. Please also note that Development will normally permit only one round of major revision.

We are aware that you may be experiencing disruption to the normal running of your lab that make experimental revisions challenging. If it would be helpful, we encourage you to contact us to discuss your revision in greater detail. Please send us a point-by-point response indicating where you are able to address concerns raised (either experimentally or by changes to the text) and where you will not be able to do so within the normal timeframe of a revision. We will then provide further guidance. Please also note that we are happy to extend revision timeframes as necessary.

© 2021. Published by The Company of Biologists under the terms of the Creative Commons Attribution License (https://creativecommons.org/licenses/by/4.0/).

Please attend to all of the reviewers' comments and ensure that you clearly highlight all changes made in the revised manuscript. Please avoid using 'Tracked changes' in Word files as these are lost in PDF conversion. I should be grateful if you would also provide a point-by-point response detailing how you have dealt with the points raised by the reviewers in the 'Response to Reviewers' box. If you do not agree with any of their criticisms or suggestions please explain clearly why this is so.

Reviewer 1

Advance summary and potential significance to field

The work of Saud and colleagues introduces a new model organism for developmental genetic studies, that has the potential to provide rapid mutagenesis screens due to their self-fertilising mode of reproduction. They were able to identify the point mutations of two mutations using a single lane of RNAseq- demonstrating the efficiency of the use of the mangrove killifish for use in forward genetic screens. A draw-back of the model is in the difficulty in injecting early embryos to rescue the phenotype. This was overcome by the authors through the use of parallel studies in medaka. The results are well presented and described. I just have a few points that must be addressed before this manuscript is suitable for publication in Development.

Comments for the author

1. The ISH in Figure 1 are images at low magnification and it is difficult though still possible, to verify the conclusions drawn in the manuscript. I assume that this is due to the fact that the body axis is situated over a large yolk sac making imaging difficult. Have the authors attempted to dissect the embryo away from the yolk and flat mount these samples?

2. In the images of btl mutants, the described bent-tail phenotype is not consistent across all images. Could the authors give numbers relating to the proportions of individuals displaying this phenotype?

3. When concluding about the regional impact on gene expression in anterior vs. posterior regions of the embryo, it might also help to comment on the morphological impact of the mutants on these regions of the body axis at these earlier developmental stages (in addition to the late stage phenotypes described in Figure 1).

4. Page. 7 line 166. The authors might also conclude that the mutants do not impact the initial balance of specification between axial and paraxial tissues but their later differentiation.

5. The rescue experiments in Figure 6 are lacking a description of n numbers for each case, and appropriate statistical tests to assay the significances in the differences observed for each condition.

6. In the discussion (line 373), the authors propose that the impact on notochord, spinal cord and paraxial mesoderm tissues is due to the lack of the maintenance of a proliferative region of progenitors in the tailbud. However, the photo-labelling experiments in Medaka instead suggest a defect in the regulation of cell movements out of the tailbud and into these tissues. Would it not then be more likely that the noto-positive region is acting as an organizer of cell movements and not proliferation? The work below demonstrates a key role for BMP in the regulation of cell movements in zebrafish:
Das, D., Jülich, D., Schwendinger-Schreck, J., Guillon, E., Lawton, A.K., Dray N., Emonet, T., O'Hern, C.S., Shattuck, M.D., and Holley, S.A. (2019).
Organization of Embryonic Morphogenesis via Mechanical Information. Dev. Cell.

Reviewer 2

Advance summary and potential significance to field

This manuscript introduces a rapid genetic analysis in the self fertilizing killifish. It is certainly an interesting model, and the authors in this paper extend the first reports of mutant identification.

© 2021. Published by The Company of Biologists under the terms of the Creative Commons Attribution License (https://creativecommons.org/licenses/by/4.0/).

They reduce the process to practice by presenting two specific examples of genes identified from the mutant screen and involved in tailbud development being identified easily, and used for further studies, which illustrate some differences in the deployment and function of the two genes between killifish and zebrafish.

Comments for the author

This manuscript introduces a rapid genetic analysis in the self fertilizing killifish. It is certainly an interesting model, and the authors in this paper extend the first reports of mutant identification. They reduce the process to practice by presenting two specific examples of genes identified from the mutant screen and involved in tailbud development being identified easily, and used for further studies.
The two genes show tailbud defects, and are characterized here with a number o fmarkers, illustrating their different effects on notochord and paraxial mesoderm development. The cloning exploited batch RNA seq of mutant and wild type phenotypes, and showed clear candidates that were proved to be noto and mesogenin.
Morpholinos were designed from the Medaka homologues and shown to phenocopy the killifish mutants in Medaka. The mutants were further characterized and each affects the other's expression, with some nice experiments with kaede lineage tracing to show the differences in segregation of tailbud tip cells between morphants and wildtype.
Overall, this is an interesting set of experiments showing the power of the self fertilizing killifish to identify and clone mutants, and in this case to also characterize the developmental behavior of cells in the mutants.

There are differences in the killifish from the zebrafish, and that is an interesting point, where one might have expected identical behavior of these genes in the different bony fishes. There is some speculation about whether this is due to different redundancy of genes, or the shape and size of the embryo, and one might have hoped that the RNA seq would at least have shown whether noto or msgn genes are duplicated differently in this species. I think this should have been addressed, since it should be easy to analyze their trinity RNA seq assemblies for duplicates. The paper is clearly written and straightforward, with perhaps more detail than needed for the identification of the candidates, which is in itself not a new approach. But this is a small point, and I overall found the paper to be a strong contribution.
Minor points From the intro "For instance, in zebrafish, the trunk cell fates are specified at gastrula stage around the blastoderm margin where the dorsal-most area gives rise to notochord, lateral side to trunk somite and spinal cord (Kimmel et al., 1990; Woo et al., 1995 Kudoh et al., 2004)." I think this is up for debate, since many cells in the Kimmel paper developed dual fates, not traditional specification test was carried out, and in general the cells only developed single germ layer fates at later gastrula stages. We now understand from single cell studies that the process of tissue "specification" is accompanied by expression of specific transcription factors, and it is debatable whether such cells are "undifferentiated". The thinking on this has been rather imprecise in the zebrafish field, and some incorrect, or at least imprecise, arguments have been made about the state of specification of cells based on what turn out to be ambiguous markers. So I think more careful language is needed, and choice of references that clearly state the conclusion made. For example I didn't see evidence from Kondoh et al 2004 that the margin is undifferentiated.

<u>Reviewer 3</u>

Advance summary and potential significance to field

The authors outline characterization of two new mutants in early patterning obtained from a forward genetic screen in the mangrove killifish, a new model for analyzing genetic regulation of development. The data is significant as this is the first identification of mutations from Kmar mutants and outline streamlined methodology for identifying through identity-by-descent.

Comments for the author

In re-review of this manuscript by Saud et al, I can appreciate the characterization presented on these mutants and limitations that arise with new model systems. However, the paper falls short,

© 2021. Published by The Company of Biologists under the terms of the Creative Commons Attribution License (https://creativecommons.org/licenses/by/4.0/).

mostly on presentation and accuracy that severely puts in question the findings they provide. The paper is poorly written, with many inaccuracies throughout. An egregious example is that Medaka is not a killifish - pointed out in last review but remains in the paper. However there are many other instances as for example stating that noto and msgn1 reciprocally interact – data which are not shown here either physically or genetically. The presentation of the filtering variants from the mRNAseq is not useful as this methodology is quite commonplace and, although details of their filtering need to be made in teh methods, the use of 3 figures to walk through this analysis is not needed. Why couldn't the mRNA seq be used to assess changes in regulation observed in these two mutants? This seems a win-win situation with using mRNA seq data over whole genome sequencing. The authors should also be careful in arguing the power of this model ("very powerful") given the data shown here. Yes mutations can be identified – however this is relatively easy these days in medaka and zebrafish as well. The lack of experimental genetic manipulation such as transgenics and Crispr-based gene editing limits Kmar use in analyzing genetic regulation. This limitation is overshadowed if Kmar specific phenotypes were studied – ones that cannot be studied in medaka or zebrafish. Kmar is an amazing animal and provides incredible opportunities to study diversity through genetic means – but this has to be put in the right light lest the community disregard it. The paper is argued by the authors to be important to demonstrate the utility of this model for forward genetics. As such, I wonder if this paper is not more suited for a techniques and resources paper as the primary developmental discoveries are limited. If so the title should be altered to reflect this shift in focus.

First revision

Author response to reviewers' comments

I believe that we have revised the manuscript to reply to all questions and requests from the reviewers, however, I also have to inform you that if further revision is requested, our possible option of capability is limited because the mutant lines that we are reporting in this manuscript are terminated during the lock down period.

Firstly, when we received reviewers' comments, our mutant fish were already old and did not spawn many eggs. Therefore to complete the revision work, we had to raise a new generation. However, a half year later, we realised (with laborious investigation) that due to a communication error between the research team and the fish facility, a new generation of the shorttail (stl) mutant strain was not raised and the stl strain was lost. Subsequently, my home office animal license was ended on June 2020 before receiving a new license. Due to this, we also had to terminate the balltail mutant line. An additional difficulty was that in the Covid19 year we were not able to do any experiments using the wild type mangrove killifish.

Despite these difficulties, we were able to add in situ markers, ntl, tbx6 and fgf8 with the last batch of embryos with one exception that we could not finish tbx6 in situ staining with stl mutant due to the loss of the line (Fig. 2). However as this marker is directly relevant to btl/msgn1 mutant but not the stl/noto pathway, therefore, we beleive that the scientific aim for analysing mutants with more marker genes has been achieved.

Please see individual replies to reviewers comments below:

Reviewer 1

"While I think the paper presents some important arguments for the use of this model as a novel tool in the field, their analysis is quite limited and unfocused. It seems as if the real finding of the paper is identifying variability in regulation of signaling to regulate tail bud organizer (through use of this fish model)."

Reply: We consider the novelties of this paper are split into two aspects: Firstly, quick identification of mutated genes using a small number of embryos within a single run of RNAseq, and secondly molecular and cellular mechanisms of tail bud development. As there are two novel

© 2021. Published by The Company of Biologists under the terms of the Creative Commons Attribution License (https://creativecommons.org/licenses/by/4.0/).

aspects with two genes to focus, it might look relatively slim in each part. We enhanced the data of both mutated gene screening (Fig,3,4,5) and mechanistic side with additional data considering the comments from reviewers (more in situ markers Fig.2).

“However, even this is not followed up as experimentation is done within a completely different fish, the ricefish medaka– putting into question the results of the genetic background sensitivity raised an underlying mechanisms for the model presented.”

Reply: We consider the mangrove killifish provides a special advantage for quick identification of mutants and mutated genes, but we also think that it is not the best model for functional analyses because of the random stage spawning of small numbers of eggs via internal self-fertilization. Therefore we added medaka for functional analyses. So far, we did not find any phenotypic differences between the killifish and rice fish species, therefore we think that the molecular and cellular mechanism of stl and btl are conserved in these species. In terms of morphology of embryos, manner of developemnt and gene homology, mangrove killifish and medaka are highly conserved compared to zebrafish. For instance, zebrafish in situ probes do not work in the Mangrove killifisha and medaka, but probes from these two species are inter-compatible.

“Quantitation of experimental analyses e.g. how many embryos showing an effect of MO or migration pattern is non-existent, leaving some lack of enthusiasm in the findings.”

Reply: The number of phenotypes of medaka MOs for noto and msgn1 are now indicated in the Figure legend for the Fig. 4I,H. Number and % of migration phenotype is indicated in the text (L288).

“The paper should be commended for bringing use of this model forward with identification of defined mutants, but the overall mix of species confounds mechanistic conclusions that can be drawn.”

Reply: As the phenotype seen in the mangrove killifish and medaka look identical, we consider the genetic and cellular mechanisms are highly conserved between these species and tried to discuss such mechanisms here. Though there are some variation in overall severity and local severity (e.g. trunk vs tail) in zebrafish, we consider the mechanisms of these genes in the tail bud development have a fundamental conservation throughout the vertebrate species.

“Kmar as a forward genetic model system: The initial focus of the paper is that of the utility of Kmar as a genetic model. There is little demonstration of this in the paper apart from that already published by Brian ring, one of the coauthors. The results section covering the character of the mutants does not connect the phenotypes with broader phenotypic class coming from the screen nor the nature of the mutations e.g. recessive, dominant? Has complementation analysis been done with this class of mutants – how is outcrossing tracked in Kmar, which has been shown to outcross in presence of males? Simple genetic analysis of the mutants is necessary to show there is utility as a genetic tool.”

Reply: It has already been reported that R109/stl and R228/btl both show Mendelian recessive patterns of phenotypic distributions in embryos (Sucar et al. 2016). Unfortunately, outcrossing is difficult and only demonstrated twice in other laboratories. In our lab, we have never been successful in crossing hermaphrodites with male fish.

“Why choice of particular mutant phenotypic class? Since this type of mutant has been screened for in many fish models, would it not leverage the strength of the model to screen for traits that are specific to Kmar and its unique adaptations?”

Reply: We appreciate that investigating unexplored mutant phenotypes may lead to novel findings. However, to establish a new species as a molecular genetics model, it would become the most solid approach if we start from mutants which show very clear and distinct phenotype with Mendelian ratio such as shorttail and balltail. TK’s research group has a long history of the investigation of the tail development (e.g. Kudoh et al. 2004, Cruz et al 2010, Finch 2010, Mourabitl 2014), therefore we felt the most comfortable way to demonstrate the simultaneous molecular characterization and RNAseq identification of mutants was to first start with experiments with these tail mutants. In

© 2021. Published by The Company of Biologists under the terms of the Creative Commons Attribution License (https://creativecommons.org/licenses/by/4.0/).

addition, these two mutants matched well each other as they showed a clear phenotype in the same region, the tail bud, therefore we were able to introduce another novelty, molecular mechanisms of the tail bud development.

“Kmar as an experimental model: The paper nicely uses comparative model to test gene function – but this reliance actually complicates the interpretation of gene function as gene function will most likely be context dependent. It is an important point that medaka is not a killifish (cyprinodontiformes), rather a ricefish of the order beloniformes. Thus, a large amount of the conclusions of conservation and function of killifishes are not correct. This lack of careful phylogenetic analysis is worrisome, especially given the reliance on the cross-taxon analysis.”

Reply: We consider overall gene function of noto/shorttail and msgn1/balltail are possibly conserved in all vertebrates. But the general severity and local severity (e.g. trunk vs tail) may have some variation due to genetic redundancy and morphological differences (e.g. yolk size). In these aspects, the mangrove killifish and medaka are very similar (similar genome size, similar embryo shape, size and speed of development). Medaka is called as killifish in some literature but as reviewer said, it is more widely accepted as ricewish, therefore the statement about “medaka as another killifish model” was removed.

“New insights into tailbud organizer: This is the strongest aspect of the presented findings. The experimental analysis showing differential regulation between trunk and rostral tail bud was interesting especially in the difference between models. Lack of quantitation e.g. even sample sizes for different analyses makes it difficult to understand how robust phenotypes are. For example, how penetrant are the shorttail and balltail mutant phenotypes? How penetrant are the MO treatments in comparison?”

Reply: As reported by Ring’s group, penetrance of stl and btl mutants follow Mendelian ratios of a recessive and fully penetrant mode of inheritance (25%). The number of morpholino phenotype is shown in the Fig.4H and I. It shows about 80% and 70% of loss of function phenotype in noto and msgn1 phenocopying a characteristic tail phenotype of stl and btl respectively. Other 20 or 30% showed severer phenotype and could not distinguish a clear phenotype (Fig.4H,I)

Reviewer 2

“It is not very clear what the initial phenotype is from the pictures, nor particularly from the in situ hybridizations. The quality of the images can be improved, perhaps by focusing on the relevant region of the fish. For example, In figure 2, there is a lot of image space wasted, and some of the features mentioned in the text are not so clear.”

Reply: The paraffin section with Hematoxillin-Eosin staining of embryo was removed as it did not provide detailed information. In the live and in situ images, the yolk part was removed and images were enlarged. Mentioned features were highlighted with arrows (Fig.1 and 2).

“In addition, the hypotheses for what is happening are based on a rather superficial number of markers, which leads the model to appear also a little unclear. There is ample work in the zebrafish that provides an excellent basis to study the phenotype here in satisfying detail, and it would seem wise to include a greater panel of markers (including the ones they cite, including brachyury and tbx6).”

Reply: Brachyury/ntl and tbx6 markers were added with one exception that tbx6 staining with noto/stl mutant was not aciheved due to the loss of the stl mutant strain. However we believe tbx6 marker is directly relevant to the function of paraxial mesoderm by msgn1/btl threfore consider that we were able to critically discuss the molecular event more in details as requested. We also added fgf8 as another marker for the tail but to gain further details of the tail bud cell fate analysis.

“I did not follow the argument in the discussion as to what was really different or similar to other bony fishes, and the discussion could be made more clear, enumerating more clearly the precedents, similarities and differences.”

© 2021. Published by The Company of Biologists under the terms of the Creative Commons Attribution License (https://creativecommons.org/licenses/by/4.0/).

Reply: The novelty of the role of noto in other cell lineage (e.g. somite mesoderm) and tail bud gene expression of msgn1 is discussed in the L309 and L324 respectively. The differential role of noto between mangrove killifish and zebrafish is discussed in the L312. Then we tried to discuss the reason of difference of phenotype between killifish/medaka and zebrafish by differential gene redundancies (L344) and/or yolk size (L379).

“For example the authors state the stl is needed to maintain tail organizer, based on gene expression, yet the tail is still there. In other animals, failure of the organizer leads to truncation. Its fair to ask what is in those tails?”

Reply: The term “Organiser” may be used as an activity for inducing the tail or organising cells in the tail. In this case, we discussed organise cells (e.g. notochord and somite cells) in gene expression and cell sorting/cell migration. As these genes are already studied in other animals with such context, we thought it was suitable to use the term of organiser. The tail organiser that induces the entire tail would be the upstream genes such as Bmp, Nodal and Fgf.

“The authors need to clarify where the polymorphisms come from. They refer to the fish as non polymorphic, and subsequently as having a small number of polymorphisms. What is the overall level of polymorphism? And presumably if the fish are indeed inbred, polymorphism is not distributed evenly over the genome, leading some regions to be inaccessible to the screening for polymorphism. Or were the lines outcrossed at some point? Indeed, I am not seeing the advantage of using an inbred line here.”

Reply: Polymorphisms possibly have been found from three ways (left over from inbred, ENU mutagenesis and sequencing error). The strong advantage of using the isogenic mangrove killifish for identifying mutated genes is discussed in Lins et al. (2018) and further by referencing that the Hon9 lineage used in this study served as the wild-type fish and the parent from prior mutagenesis to create the mutants described here (L413).

The fact that we identified single mutations from two mutants without using mapping information would be a strong proof that this approach is extremely effective. Though it is not included, we have another mutant, no-fin-ray, from this line, we sequenced 3 mutant embryos, 3 siblings, and 3 wild type (only 9 embryos total) and identified the mutated gene, edaradd (cytoplasmic protein in the EDA/EDAR pathway). We are confident that this approach is extremely useful, easy and not comparable to other systems (e.g. zebrafish, medaka and mice) for identifying a mutated gene from a mutant.

“Perhaps the images could focus just on the embryonic and not extraembryonic parts of the specimens, and show them at higher magnification.”
Reply: The images of embryos were enlarged by removing the yolk part (Fig.2, Fig. 6)

“In fig 4, the text refers to A and M instead of J and M for the in situ pictures
Again the images do not focus on the relevant region of the embryo, and the stain is therefore indistinct. It would also be useful to see earlier stages to see where the gene is expressed as the trunk is being laid down.”

Reply: Earlier stage of WT embryos stained with noto and msgn1 probes were added (gastrula and early somite stage) (Fig.7A-D) showing consistent expression of noto and msgn1 in the prospective axial and paraxial domains of the tail bud.

“Being outside the fish field, I found it awkward that the authors do not switch their nomenclature to the official gene identity. It makes the work harder to follow, since one has to remember the identities of stl and btl, at the same time the genes are referred to as noto and msgn. Also having grown up in the era of floating head etc, it might be useful to remind the reader that noto used to be flh (the manuscripts they refer to of course used the old terminology).”

Reply: Thank you for the suggestion. In the later half after the when stl and btl are identified as mutants of noto and msgn1 respectively, the mutants are now referred to as stl/noto or btl/msgn1 mutant.

© 2021. Published by The Company of Biologists under the terms of the Creative Commons Attribution License (https://creativecommons.org/licenses/by/4.0/).

“The results of the lineage tracing of the tailbud are clear and well presented. They provide some of the best arguments in favor of the model. But I am not clear on what is excluded, or different from zebrafish.”

Reply: This data is consistent with the data from mice work therefore we did not discuss it with a strong novelty. But our data show much enhanced and clearer result than the one in zebrafish possibly due to lower redundancy and this is discussed in the section L355-377)

Reviewer 3

“The images of ISHs in figures 2 and 4 are very difficult to see. It would help to have magnified insets for each image to be able to see in more detail the alterations in gene expression that the authors are pointing to.”

Reply: The image is magnified with removing the yolk part.

“The conclusions relating to te role that noto and mesdogenin play on the development of the spinal cord are not sufficiently supported by the data presented. The only indication of this is the ISH for Sox3 performed in the mutants and as the authors point out themselves, this is difficult to visualise in the btl mutant due to the bent tail phenotype. While the reduced expression in the stl mutants are indicative of a role in spinal cord development, the ISH is weaker across the whole AP axis suggesting that this might simply be due to a weaker staining of this particular sample. I would suggest that the reviewers deal with these results with more care in their introduction and to remove the spinal cord as a part of their model.”

Reply: As suggested by the reviewer, we removed the model figure and simplifies the involvement of noto in the spinal cord.

“Throughout the manuscript there is little or no indication about the numbers of embryos that were examined in each experiment, and the percentage of effected embryos that they saw in each case. It is especially important to clarify this where conclusions are drawn on the intensity of gene expression observed by ISH, as slight variations in staining duration can lead to very different results.”

Reply: Mutant phenotype is highly consistent in all mutant embryos in morphology and gene expression. Each mutant with individual markers were examined with 5 embryos (main text, Line 173). Other numubers are also added as explained in the reply to reviewer-2.

“Further quantification is required alongside the experiments shown in Figure 5. How many embyos were analysed for each experiment, and how reproducible are these results? Ideally the authors would show multiple examples of each experiment as supplementary data, but at the very least numbers need to be given in the main text.”

Reply: Now, number of samples and consistency of the result within a treatment group is discussed in the result section (n=5, L288)

“5) In the discussion the authors compare their results to mutants in zebrafish embryos. One of the strengths of the work is the comparative aspect as the presentation of their model organism has the potential to generate new and interesting insights into how gene function as altered during the evolution of the vertebrate body plan. However, the authors miss several important papers relating to mesogenin and noto function in the zebrafish that should be included as part of their discussion. These include:”

Reply: The papers listed by the reviewer is added and discussed except the Stemple et al. Odenthal and Stemple papers publsihed similar work (identification of notochord mutant zebrafish lines) at the same time and only the former paper included the noto mutant line.

© 2021. Published by The Company of Biologists under the terms of the Creative Commons Attribution License (https://creativecommons.org/licenses/by/4.0/).

Second decision letter

MS ID#: DEVELOP/2021/199675

MS TITLE: Molecular mechanisms of embryonic tail development in the self-fertilizing mangrove killifish, Kryptolebias marmoratus

AUTHORS: Hussein A Saud, Paul A O'Neil, Yosuke Ono, Bas Verbruggen, Ronny van Aerle, Jaebum Kim, Jae-Seong Lee, Brian C Ring, and Tetsuhiro Kudoh
ARTICLE TYPE: Research Article

I am happy to tell you that your manuscript has been accepted for publication in Development, pending our standard ethics checks.

Reviewer 1

Advance summary and potential significance to field

The work of Saud and colleagues introduces a new model organism for developmental genetic studies, that has the potential to provide rapid mutagenesis screens due to their self-fertilising mode of reproduction. They were able to identify the point mutations of two mutations using a single lane of RNAseq- demonstrating the efficiency of the use of the mangrove killifish for use in forward genetic screens. A draw-back of the model is in the difficulty in injecting early embryos to rescue the phenotype. This was overcome by the authors through the use of parallel studies in medaka.

Comments for the author

The authors have now satisfactorily addressed my major concerns.

Minor concerns
The resolution of the table text should be improved prior to publication. In addition several of the figures should be re-organised to make the best use of space.

© 2021. Published by The Company of Biologists under the terms of the Creative Commons Attribution License (https://creativecommons.org/licenses/by/4.0/).
